# Report on a case of liver-originating malignant melanoma of unknown primary

**DOI:** 10.1515/biol-2022-0750

**Published:** 2023-10-23

**Authors:** Zheng Yuan, Hai-yan Guo, Wei-ting Lu, Yao-hui Wang, Jing He, Fan Zhang, Jun-yong Che, Fei Qiao

**Affiliations:** Department of Infectious Diseases, Affiliated Hospital of Nanjing University of Chinese Medicine, No. 155 of Hanzhong Road, Qinhuai District, Nanjing 210000, China; Department of Pathology, Affiliated Hospital of Nanjing University of Chinese Medicine, Nanjing 210000, China

**Keywords:** diagnosis, liver, malignant melanoma, unknown primary

## Abstract

Malignant melanoma (MM) frequently occurs in the skin or mucosa, whereas malignant melanoma of unknown primary (MUP) is diagnosed in patients with lymph nodes or visceral organs as the site of origin, where it is challenging to detect the primary lesion by comprehensive examination. MUP is possibly related to the spontaneous regression of the primary lesion. In addition, primary hepatic melanoma (PHM) usually refers to the primary MM occurring in the liver, with no typical primary lesions and no manifestations of tumor metastasis. A 61-year-old male patient with liver as the site of origin was diagnosed with MM by Melan-A, HMB-45, and S-100 immunohistochemistry staining of liver biopsy tissue. Based on a comprehensive examination, no basis was found for melanoma in sites such as the skin, mucosa, five sense organs, brain, digestive tract, respiratory tract, or genitalia, and the patient was subsequently diagnosed with MUP. MMs require a comprehensive inspection, beginning with the liver, to search for the primary lesion; if the primary lesion is not found, the possibility of PHM or MUP should be considered.

## 1 Introduction

Malignant melanoma (MM) is a strongly aggressive cancer that occurs primarily at the junction of epidermis and dermis, or in regions abundant in melanocytes such as the skin, mucosa, ciliary body of the eye, iris, choroid, and meninges [1]. With more than 95% of cases of MM originating in the skin, skin MM metastases occur most frequently in the liver, lungs, bones, and brain. When a patient is diagnosed with metastatic MM, the primary lesion should be identified in time, but there are still some cases of melanoma of unknown primary (MUP) that involve lymph nodes or visceral organs and are not located in the skin [2]. In this study, a patient with MUP manifesting primarily as liver lesions was diagnosed and treated, as reported below.

## 2 General information

The patient was a 61-year-old male retiree who was in excellent health and had no history of skin and eye diseases or related treatment, no toxic exposure, no addiction to tobacco or alcohol, and no family history of hepatocellular carcinoma.

### 2.1 Clinical features

The chief complaint was abdominal distension accompanied by loss of appetite and weight loss for more than a month. Symptoms included abdominal distention, poor appetite, nausea, weakness, and progressive weight loss. On physical examination, no obvious melanocytic nevi were discovered on the skin and mucosa of the entire body. Palpation revealed that the site, a width of four transverse fingers below the xiphoid process of the liver, was harder, hypersensitive, and nodular.


**Informed consent:** Informed consent has been obtained from all individuals included in this study.
**Ethical approval:** The research related to human use complied with all the relevant national regulations, institutional policies and is in accordance with the tenets of the Helsinki Declaration, and has been approved by the Ethics Committee of Affiliated Hospital of Nanjing University of Chinese Medicine.

### 2.2 Blood test

Blood routine examination was basically normal; liver function: GOT 50 U/L (10–50 U/L), GPT 69 U/L (10–40 U/L), Glb 35.0 g/L(35–52 g/L), Alb/Glb 1.03, AKP 859 U/L (40–130 U/L), GGT 623 U/L (8–61 U/L), TBil 42.60 µmol/L (<24 µmol/L), DBil 27.60 µmol/L (<5 µmol/L), ChE 2126 U/L (5,900–12,220 U/L); PCT: 2.10 ng/mL (<0.046 ng/mL); HBsAg, HBcAb, and HCV antibodies were negative; tumor markers: CA19-9 49.2 IU/mL (<37 IU/mL), FER 724 ng/mL (23.9–336 ng/mL), AFP 1.34 IU/mL (<10.9 ng/mL).

### 2.3 Imaging examination

**Figure 1:**
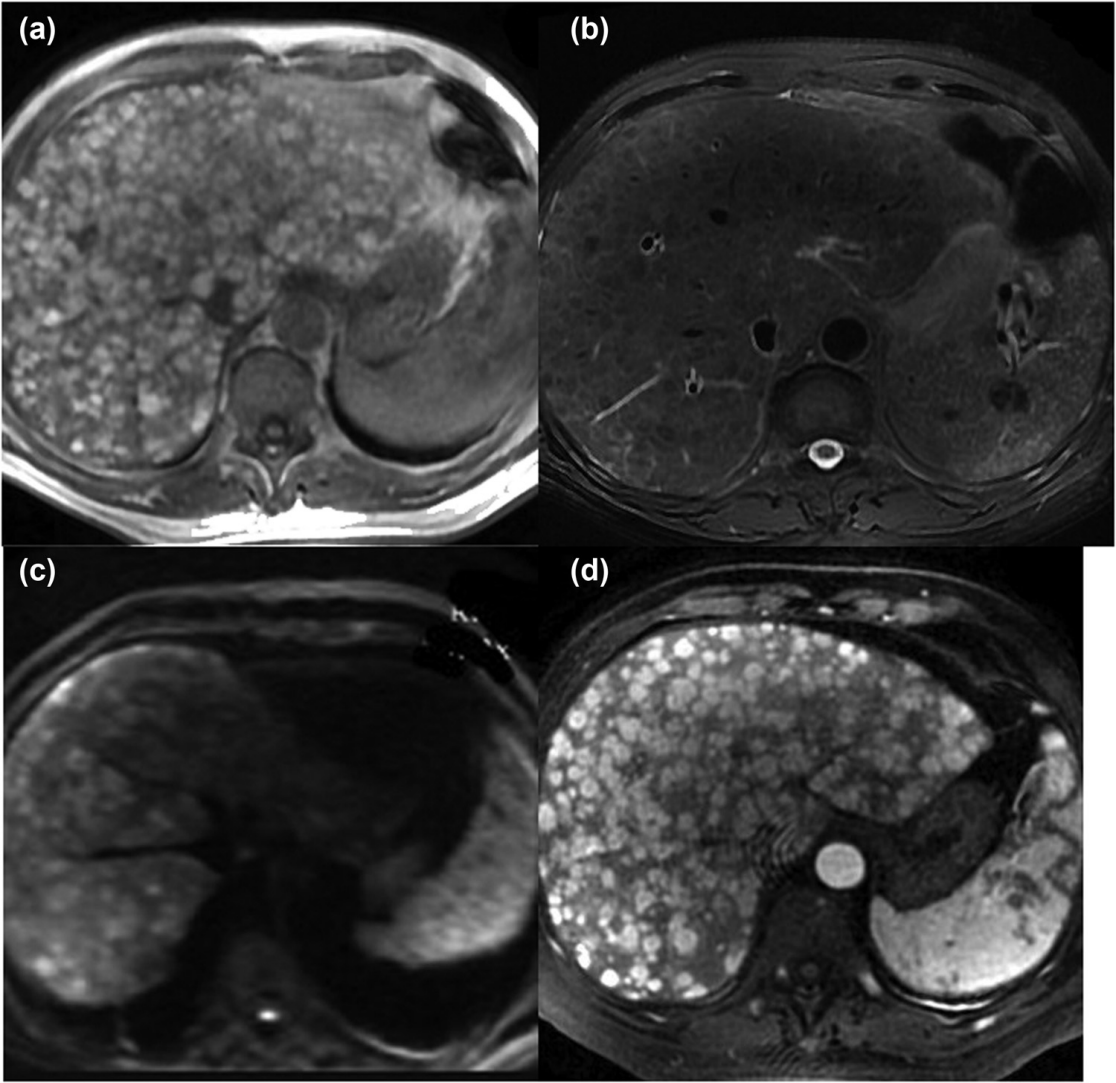
MRI shows diffuse space-occupying lesions of the liver, T1WI high signal (a), T2WI low signal (b), DWI high signal (c), and unequal enhancement of foci in the arterial phase (d).

**Figure 2:**
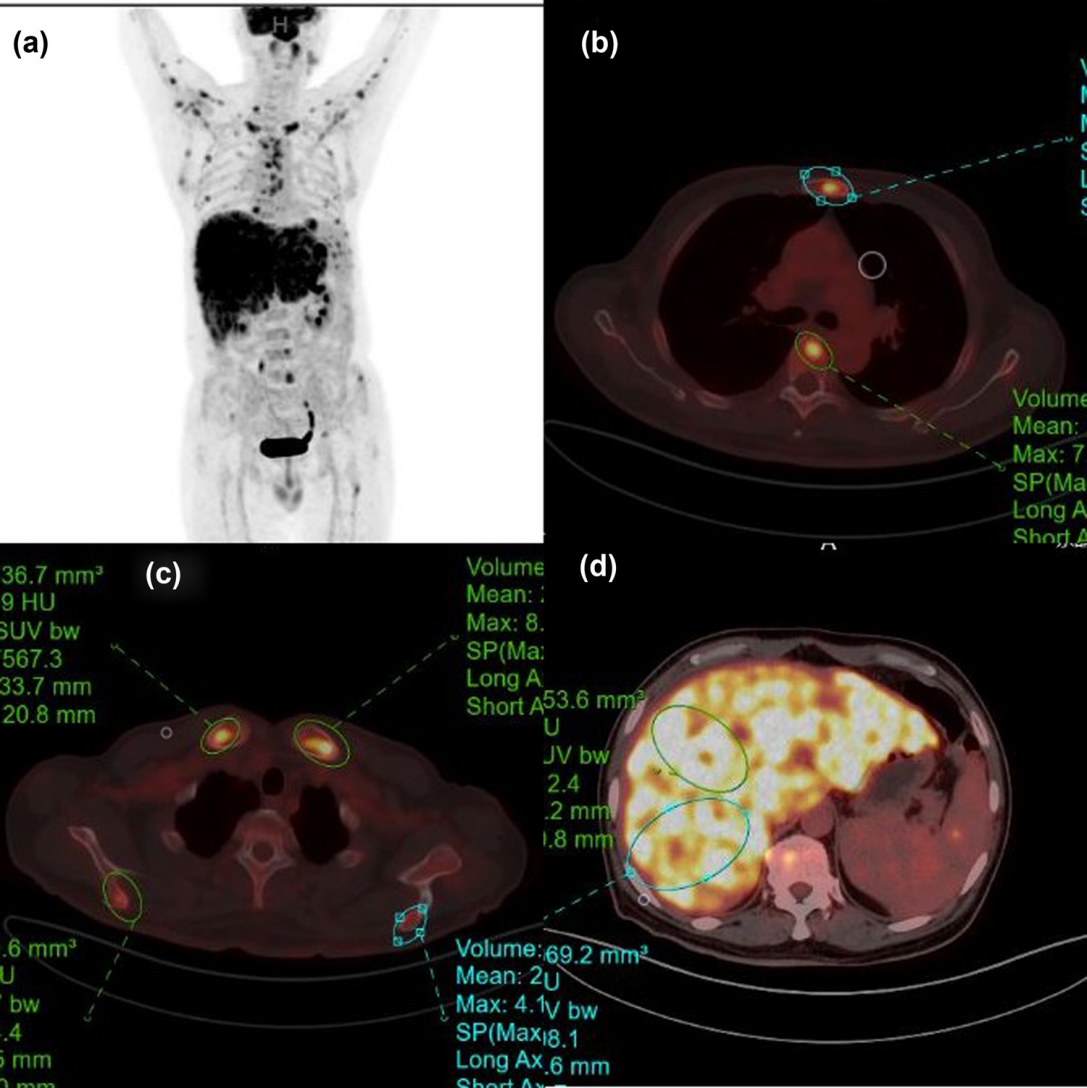
PET-CT shows an increase in the SUV uptake value in multiple parts of the body (a), such as the liver and spleen (d), sternum and vertebrae (b), and other parts of the bone (c).

Abdominal computed tomography (CT) enhancement suggests hepatomegaly, diffuse space-occupying lesions of the liver, uneven enhancement in the arterial phase, and splenomegaly; abdominal MRI reveals diffuse space-occupying lesions of the liver (Figure 1); positron emission tomography/CT (PET/CT) reveals diffuse modules in the liver, multiple nodules in the spleen, multiple bone density changes, slightly larger retroperitoneal lymph nodes, multiple nodes in the left parotid gland and neck and the right cardiophrenic angle region, nodules in both arm muscles, and fluorodeoxyglucose uptake enhancement in all these areas, and thus a malignancy was considered (Figure 2).

### 2.4 Diagnosis

**Figure 3:**
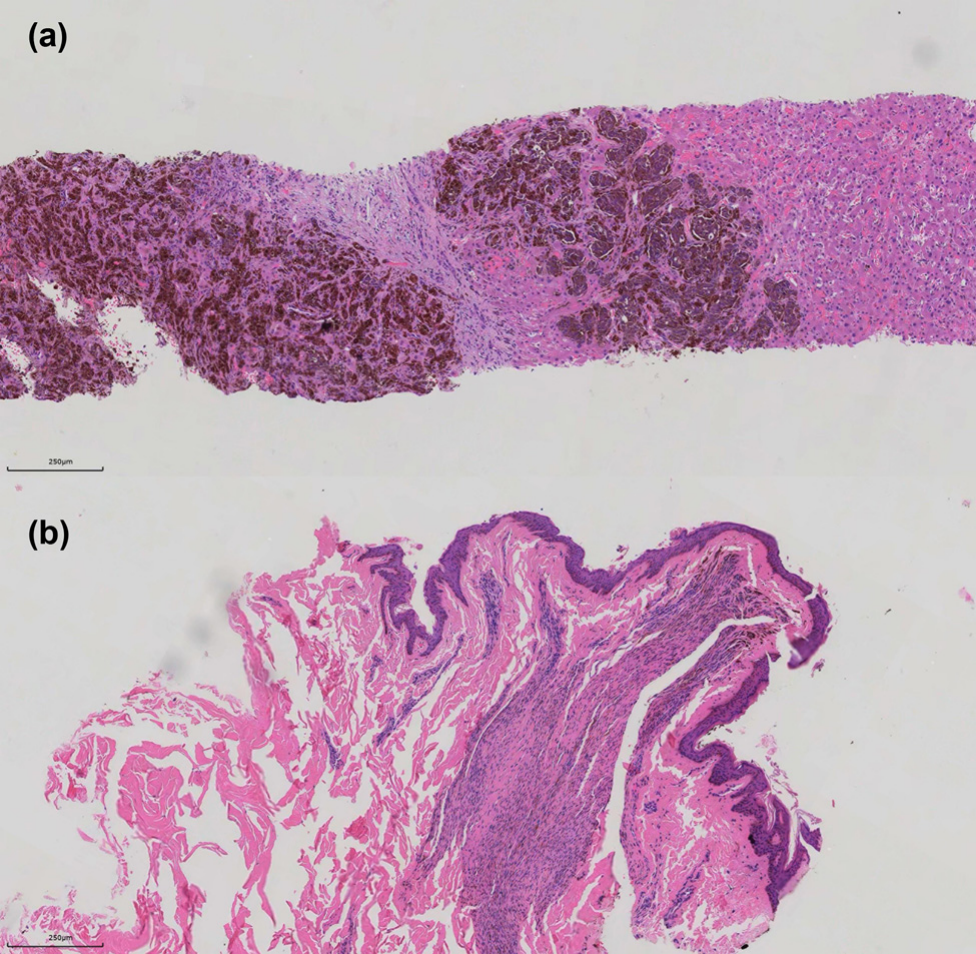
Liver histopathology (HE ×100) reveals nestlike cellular infiltration, accompanied by more pigmentation and nuclear enlargement with evident heteromorphism (a); the histopathology of pigmented nevi posterior to the left scapula (HE ×100) indicates benign cutaneous nevus (b).

Pathology of liver biopsy tissue: appearance: black, under the microscope: a malignancy with pigmentation (Figure 3); immunohistochemical (IHC) of paraffin section: Melan-A (++), HMB45 (++), S-100 (++), P16 (scattered+), Ki67 (about 25%), CgA (−), Syn (−), CK-P (−), BRAF (V600E) (+), indicating MM. Gastroenterological endoscopy results were negative. The ophthalmology, otorhinolaryngology, and dermatology consultations revealed no primary lesion at the corresponding site. Malignant lesions were excluded by biopsy pathology after a thorough physical examination, which revealed an oval, blue-gray pigmented nevus of approximately 0.3 cm in length and diameter on the skin posterior behind the left scapula (Figure 3). The patient was finally diagnosed with MUP with multiple hepatic, splenic, bone, and muscular metastases, based on the above data, and no basis was found for melanoma in such sites as the skin, mucosa, five sense organs, brain, digestive tract, or genitalia.

### 2.5 Treatment and prognosis

The patient was treated once with 200 mg of pembrolizumab monoclonal antibody, followed by target therapy. At a 5 month follow-up, the patient complained of a modest improvement in abdominal distension and weakness but continued to have a poor appetite, and imaging re-examination suggested no obvious changes in intrahepatic lesions.

## 3 Discussion

MUP, with an incidence of approximately 3% in MM, is defined as metastatic melanoma detected under the skin and in lymph nodes or other visceral organs, with no obvious indications of the primary lesion [3,4,5]. DasGupta et al. proposed the diagnostic criteria for MUP in 1963 [6] based on which the following cases were excluded: (1) patients who did not undergo a comprehensive physical examination, including anus, genitalia, and eye examinations; (2) patients with a history of orbital dissection; (3) patients who underwent surgical or non-surgical procedures for birthmarks, freckles, chronic paronychia, or skin lesions without undergoing pathological examinations; and (4) patients who developed enlarged lymph nodes and scars in the skin with lymphatic drainage. In this study, the patient had no history of dermatology or ophthalmology diseases or treatments; no clear skin or mucosal lesions were identified during a comprehensive physical examination; the gastrointestinal source was excluded using a gastroenterological endoscope; and eye lesions were excluded using an ophthalmic consultation. The patient fulfilled the diagnostic and exclusion criteria for MUP.

Regression of the primary focus is the most likely reason for the pathogenesis of MUP to remain unclear [7]. The frequency of complete regression of primary skin MM is assumed to range between 3.7 and 8.7% [8]. Males are more susceptible to regression than females [9]. There is an argument that males are more likely to ignore early primary melanoma in the skin before its regression and metastasis. Therefore, it is necessary to identify the MM in which the primary focus has completely regressed, and thoroughly check the skin of all patients diagnosed with MM [10]. Some studies have recommended the following MUP examinations [11]: comprehensive examination of skin and mucosal surfaces; ophthalmologic examination, otorhinolaryngologic examination (nasal cavity); gastrointestinal endoscopy (oral cavity, esophagus, anus); gynecologic examination (vulva, vagina, cervix, uterus); brain, neck, chest, and abdominal CT scanning; other possible sites include the gallbladder and ovaries.

Initial consideration was given to the rare diagnosis of primary hepatic melanoma (PHM), for which only a few reports exist [12,13]. Melanophores originate from neural crest cells, a transiently migrating population of stem cells [14]. Extracutaneous MM is believed to be caused by melanoblasts accompanying the mucosa, stroma, and neurovascular bundles or neuroectodermal rest cells migrating to these sites [15]. Therefore, primary visceral MM is theoretically valid. Due to the absence of unified diagnostic criteria for PHM, some scholars argue that the diagnostic criteria [16] should include the three primary conditions and one secondary condition listed below. Three primary conditions: (1) support from hepatic histopathology; (2) absence of evidence of melanoma in other sites; and (3) no history of skin lesions of unknown type or eye surgery. One secondary condition: a single lesion or multiple lesions with at least one lesion larger than 5 cm in diameter. The patient in this study met the above primary diagnostic conditions, but he failed to meet the secondary condition because he had multiple diffuse space-occupying lesions of the liver with a diameter of less than 5 cm and no primary focus. At the same time, the imaging findings of this patient do not conform to the common features of PHM: single or multiple cystic-solid space-occupying lesions in the liver [17]. Therefore, the patient could not be diagnosed with PHM.

MRI is the preferred imaging technique for detecting the liver and spleen metastases of MM [18]. In addition, studies indicate that systemic MRI is 100% sensitive, 100% specific, and 100% accurate in the diagnosis of hepatic metastases of melanoma [19]. Normal MM that contains melanin may exhibit a paramagnetic effect during an MRI examination, which may shorten T1 and T2 relaxation times and display a high T1WI signal and a low T2WI signal [20]. The patient in this investigation exhibited this feature. Thus, the MRI examination is extremely beneficial for the recognition and diagnosis of MM lesions of the liver. Of the 24 MUP patients reported, 11 patients underwent PET that showed no primary focus, possibly because the primary focus was too small or had spontaneously regressed [21]. This suggests that PET is less effective in discovering the primary tumor site of melanoma but is useful for detecting metastatic lesions and developing treatment regimens.

In terms of prognosis, the mean survival of MUP patients with lymphatic metastasis is 24–165 months, and that of MUP patients with visceral metastasis is 3–13.2 months [3], suggesting that the latter have shorter overall survival [22]. In terms of treatment, patients with MUP should be actively treated according to the same standards as patients with metastatic disease whose primary disease has been completely treated, and resectable lesions should be surgically removed as much as possible. In the event of lymphatic metastasis, lymph node dissection should also be conducted [21]. However, the patient in this study could not receive surgical resection due to hepatic diffuse metastasis with metastases in other sites and was treated primarily with immunotherapy and targeted therapy, combined immune checkpoint inhibitors of cytolytic T lymphocyte-associated antigen 4 and programmed death 1 could be considered as a therapeutic regimen [23].

## 4 Conclusion

MM originating from the liver necessitates the accumulation of a comprehensive medical history, a thorough physical examination, and a corresponding auxiliary examination in order to search for the primary lesion. If the primary lesion cannot be identified, PHM or MUP should be considered.
